# Supplementary material for: Asynchronous magnetic resonance elastography: Shear wave speed reconstruction using noise correlation of incoherent waves
Source: Magn Reson Med. 2022 Oct 27;89(3):990–1001. doi: 10.1002/mrm.29502 (PMC9792433; doi:10.1002/mrm.29502)
Supplement: Supplementary file 1 — DATA S1 MATLAB code used to generate the numerical simulations of Figure 1 [file MRM-89-990-s002.zip › k-Wave/helpfiles/logCompression.html]

logCompression :: Functions (k-Wave)


# logCompression

Log compress an input signal.

## Syntax

```
signal = logCompression(signal, a)
signal = logCompression(signal, a, normalise)
```

## Description

`logCompression` compresses the input signal using the expression `signal = log10(1 + a * signal) ./ log10(1 + a)`.

## Inputs

|  |  |
| --- | --- |
| `signal` | input signal |
| `a` | compression factor |

## Optional Inputs

|  |  |
| --- | --- |
| `normalise` | Boolean controlling whether the maximum of the input signal is normalised to unity before compression (default = false). If set to true, the original magnitude is restored after compression. |

## Outputs

|  |  |
| --- | --- |
| `signal` | log compressed signal |

## See Also

`log10`
